# Motility-activating mutations upstream of *flhDC* reduce acid shock survival of *Escherichia coli*

**DOI:** 10.1128/spectrum.00544-24

**Published:** 2024-04-23

**Authors:** Kilian Schumacher, Djanna Braun, Karin Kleigrewe, Kirsten Jung

**Affiliations:** 1 Faculty of Biology, Microbiology, Ludwig-Maximilians-Universität München, Martinsried, Germany; 2 Bavarian Center for Biomolecular Mass Spectrometry (BayBioMS), Technical University of Munich, Freising, Germany; Lerner Research Institute, Cleveland, Ohio, USA

**Keywords:** motility, flagella, acid resistance, acid shock survival, insertion sequences

## Abstract

Many neutralophilic bacterial species try to evade acid stress with an escape strategy, which is reflected in the increased expression of genes coding for flagellar components. Extremely acid-tolerant bacteria, such as *Escherichia coli*, survive the strong acid stress, e.g., in the stomach of vertebrates. Recently, we were able to show that the induction of motility genes in *E. coli* is strictly dependent on the degree of acid stress, i.e., they are induced under mild acid stress but not under severe acid stress. However, it was not known to what extent fine-tuned expression of motility genes is related to fitness and the ability to survive periods of acid shock. In this study, we demonstrate that the expression of FlhDC, the master regulator of flagellation, is inversely correlated with the acid shock survival of *E. coli*. We encountered this phenomenon when analyzing mutants from the Keio collection, in which the expression of *flhDC* was altered by an insertion sequence element. These results suggest a fitness trade-off between acid tolerance and motility.

**IMPORTANCE:**

*Escherichia coli* is extremely acid-resistant, which is crucial for survival in the gastrointestinal tract of vertebrates. Recently, we systematically studied the response of *E. coli* to mild and severe acidic conditions using Ribo-Seq and RNA-Seq. We found that motility genes are induced at pH 5.8 but not at pH 4.4, indicating stress-dependent synthesis of flagellar components. In this study, we demonstrate that motility-activating mutations upstream of *flhDC*, encoding the master regulator of flagella genes, reduce the ability of *E. coli* to survive periods of acid shock. Furthermore, we show an inverse correlation between motility and acid survival using a chromosomal isopropyl β-D-thio-galactopyranoside (IPTG)-inducible *flhDC* promoter and by sampling differentially motile subpopulations from swim agar plates. These results reveal a previously undiscovered trade-off between motility and acid tolerance and suggest a differentiation of *E. coli* into motile and acid-tolerant subpopulations, driven by the integration of insertion sequence elements.

## INTRODUCTION

Bacteria colonize habitats such as the human stomach and acidic soils, which have a low pH (1, 2). In these environments, protective mechanisms against acidity are crucial to ensure the growth and/or survival of neutralophilic bacteria. Particularly, *Escherichia coli* exhibits substantial tolerance to acidity and has a corresponding infective dose, which is several orders of magnitude lower in comparison to other enteropathogens (3). Pathogenic *E. coli* strains, as well as non-pathogenic K-12 lab strains, survive at extremely low pH (<2.5) (4). This is crucial for pathogenic strains in order to endure the low pH of the stomach, which constitutes the major bactericidal barrier of the human gastrointestinal tract (5, 6). As a consequence, a multitude of defensive strategies have evolved in *E. coli* in order to adapt to such extreme conditions, including H^+^-consuming acid resistance systems, acid shock proteins, restrained membrane permeability by altered fatty acid compositions, proton pumps, and many others (3, 7–9).

At moderate acid stress, many neutralophilic bacterial species use an escape strategy to avoid acidity, which is reflected by the increased expression of flagellar genes (10, 11). Accordingly, our RNA-Seq and Ribo-Seq data indicated that chemotaxis and motility genes were induced only under mildly acidic (pH 5.8) but not under severely acidic conditions (pH 4.4) (12). Most of the pH-affected motility genes belonged to the FlhDC regulon (13, 14). FlhD and FlhC form heterohexamers, and the complex acts as the master regulator of flagella synthesis and motility in *E. coli* (15–17). Genes regulated by FlhDC involve class II flagellar operons encoding basal body components, flagella export systems, the alternative sigma factor FliA, and non-flagellar operons (14, 18, 19). The *flhDC* operon is under the transcriptional control of a large number of transcription factors and small RNAs, which implement environmental cues such as osmolarity, synthesis of fimbriae, catabolite repression, and quorum sensing (20–23).

Notably, several motility-activating mutations in the promoter region of *flhDC* have been described. These include insertion sequence (IS) elements and point mutations, which disrupt the binding sites of transcriptional repressors like OmpR or LrhA (24–27). The presence of such mutations varies among various *E. coli* K-12 strains and affects motility rates due to consequently enhanced *flhDC* expression levels (24–27). Strikingly, motility-activating mutations in the *flhDC* regulatory region have also been detected in several single-gene knockout mutants of the Keio collection (28).

In our previous study, we noted significantly increased transcript and ribosome footprint levels for FliA and FlhDC at pH 5.8 (12). However, the highest increase among all annotated transcription factors in *E. coli* was found for the IclR-type regulator MhpR at pH 4.4 (12). Moreover, an *mhpR* mutant obtained from the Keio collection (29) showed the lowest survival rate among all tested knockout mutants of crucial acid resistance regulators under acid shock (12). MhpR regulates the expression of an operon involved in the degradation of cinnamic acid derivatives such as phenylpropionate (PP), 3-(2,3-dihydroxyphenyl)propionate (DHPP), and 3-hydroxyphenylpropionate (3HPP) (30, 31). These aromatic compounds can bind individually or synergistically to MhpR and stimulate the interaction of MhpR with its operator sequence in the *mhpABCDFE* promoter region (32). Upon induction of the *mhpABCDFE* genes, 3-HPP and PP can be converted first to DHPP, which is ultimately degraded to intermediates of the citric acid cycle (30, 33). 3HPP and PP are commonly ingested through the uptake of plant material. For instance, several hydroxycinnamic acid derivatives are found to large extents in blueberries and huckleberries (34) and occur as secondary metabolites in other plants (35). However, the contribution of MhpR to acid resistance in *E. coli* is unclear.

In this study, we show that the reduced survival rate of an *mhpR* mutant from the Keio collection is caused by the integration of an IS5 element in the promoter region of *flhDC* and not by *mhpR* itself. RNA-Seq and soft agar swim assays confirmed that this mutant is hypermotile. The presence or absence of IS elements in *flhDC* promoter regions of different *E. coli* strains affected not only motility but also survival under acid shock. Using a strain with a chromosomal isopropyl β-D-thio-galactopyranoside (IPTG)-inducible *flhDC* promoter, we found an inverse correlation between motility and the ability to survive acid shock periods. These results indicate that induced expression of flagellar components, which is beneficial in mildly acidic environments, is detrimental for *E. coli* under severe acid stress and requires tight regulation of FlhDC target genes.

## RESULTS

### Examination of a putative role of MhpR under severe acid stress

**Fig 1:**
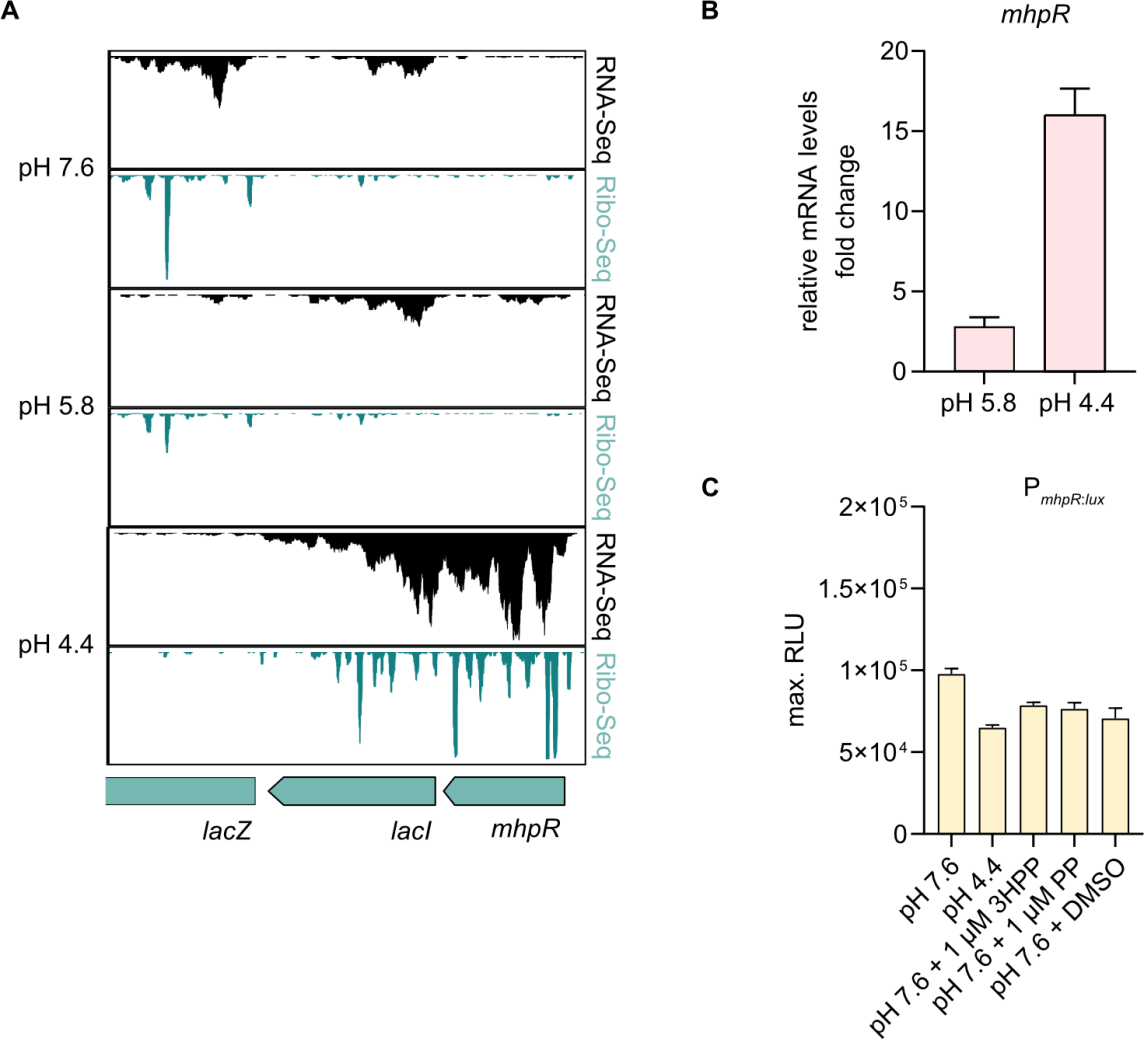
*mhpR* is post-transcriptionally upregulated under severe acid stress. (**A**) JBrowse2 (36) screenshots of read coverage from Ribo-Seq (green tracks) and RNA-Seq (black tracks) libraries at pH 7.6, 5.8, and 4.4. Schematic illustrations below indicate the genomic locations of *mhpR* and adjacent genes. Ribo- and RNA-Seq data were obtained from Schumacher et al. (12). (**B**) Verification of increased *mhpR* mRNA levels under acid stress using RT-qPCR. Cells were cultivated as described by Schumacher et al. (12). Fold change values were calculated relative to pH 7.6 and normalized using *secA* as a reference gene. Error bars indicate the standard deviation of three independent biological replicates (*n* = 3). (**C**) Luciferase-based promoter assay. *E. coli* MG1655 wild-type cells were transformed with the plasmid pBBR1-MCS5-P*_mhpR_*_:*lux*_ and grown in LB medium (pH 7.6) until OD_600_ = 0.5. The pH of the medium was then either adjusted stepwise to 5.8 and to pH 4.4, or 1 mM 3HPP, 1 mM PP, or dimethyl sulfoxide (DMSO) was added. Luminescence and growth were determined every 10 min in microtiter plates using a CLARIOstar plus plate reader (BMG Labtech). Data are reported as relative light units (RLUs) in counts per second per OD_600_, with maximal RLU shown. All experiments were performed in biological replicates (*n* = 3), and error bars represent standard deviations of the mean.

We have previously detected enriched transcript and ribosome occupancy levels of *mhpR* in *E. coli* K-12 MG1655 at pH 4.4 (Fig. 1A) (12). To verify whether *mhpR* is upregulated under severe acid stress and to distinguish whether the regulation occurs at the transcriptional or post-transcriptional level, we examined *mhpR* mRNA levels and promoter activity under acid stress and non-stress conditions in *E. coli* MG1655. qRT-PCR analysis revealed that *mhpR* transcript levels increased only slightly at pH 5.8, but 15-fold at pH 4.4 compared to pH 7.6 (Fig. 1B).

Next, we constructed a luciferase-based promoter-activity reporter plasmid (pBBR1-MCS5-P*_mhpR_*_:*lux*_). The *mhpR* promoter was constitutively active regardless of the extracellular pH and the presence of PP or 3HPP (Fig. 1C). This implies that the *mhpR* promoter is not affected by acidification, which is in line with previous findings (31). These results suggest that the upregulation of *mhpR* under severe acid stress is mediated by a post-transcriptional mechanism.

We then tested whether the described activation of a catabolic pathway for cinnamic acid derivatives (30–32) is related to MhpR under acid stress. However, this hypothesis could be neglected as we did not detect any activation of the *mhpABCDFE* promoter at pH 4.4 (Fig. S1). We also investigated whether cinnamic acid derivatives (3HPP, PP, and DHPP) are present in the LB medium and whether their abundance increases in a pH-dependent manner. For this purpose, we analyzed the LB medium and sterile-filtered supernatants of cultures grown at pH 7.6, 5.8, or 4.4 by LC-MS (see Materials and Methods for details). Neither PP, DHPP, nor 3HPP were detectable in any of the analyzed samples (data not shown). Thus, we conclude that both the enzymes responsible for 3HPP, PP degradation (MhpA-F) and the corresponding substrates for the pathway are not present in pH-neutral and acidified LB media. In summary, MhpR-induced catabolism of 3HPP and PP does not play a role under acid stress adaptation.

### Phenotypic characterization of *E. coli mphR* mutants with different genetic backgrounds

**Fig 2:**
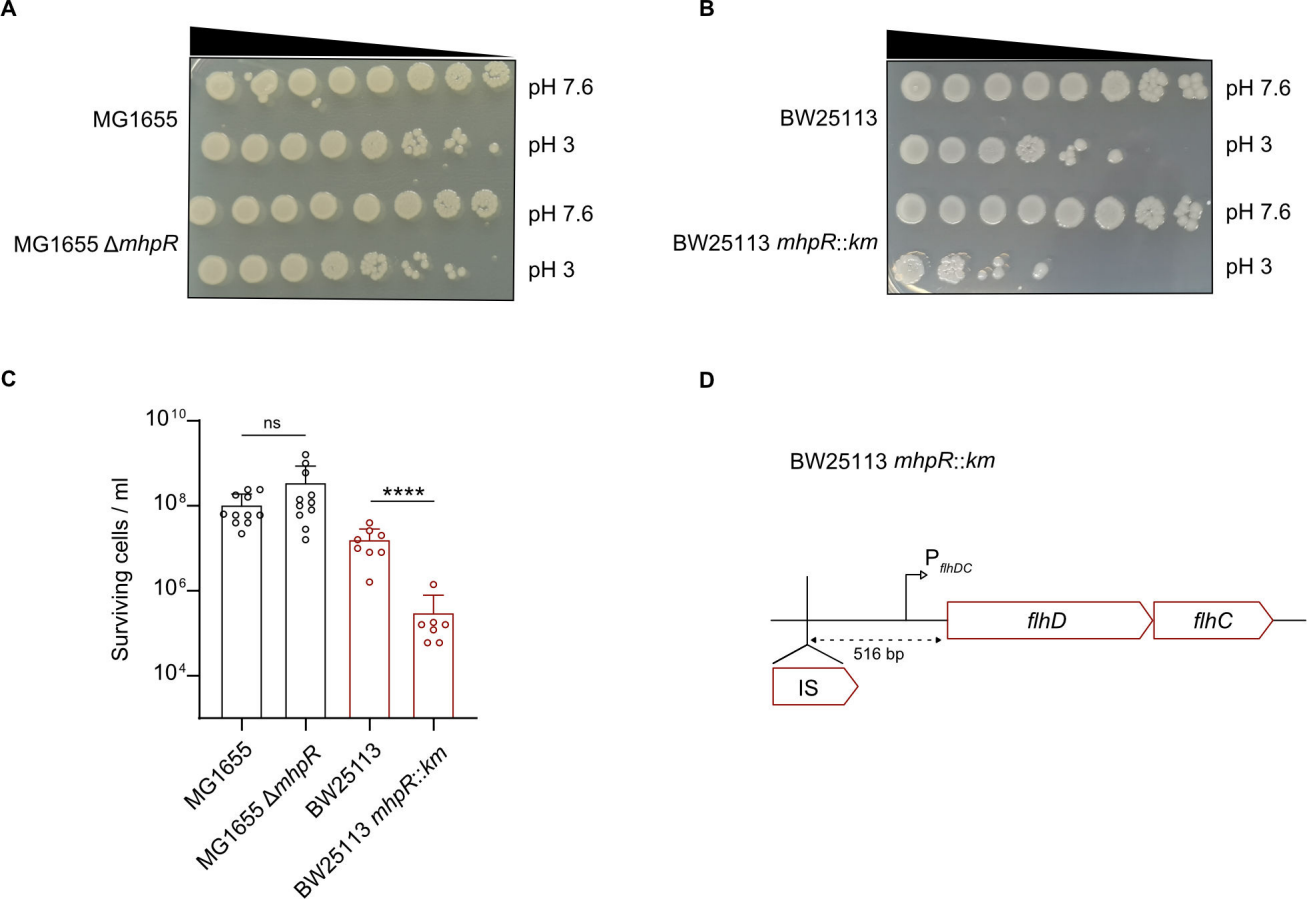
Survival under acid shock is exclusively affected in an *mhpR* mutant from the Keio collection. (**A and B**) Acid shock assays to evaluate the survival of *E. coli* MG1655 and MG1655 Δ*mhpR* (**A**) and BW25113 and BW25113 *mhpR::km* (**B**) at pH 3. Cells were grown in LB pH 7.6 to OD_600_ = 0.5 and cell numbers were adjusted to 10^9^/mL. Cultures were split and then either grown at pH 7.6 or stepwise stressed (15 min pH 5.8 and 15 min pH 4.4) before being exposed to LB pH 3 for 1 h. Cultures were serially diluted by a factor of 10 in 1× PBS and plated on LB agar plates. Images were taken after overnight incubation. (**C**) Quantitative assessment of acid shock survival of *E. coli* MG1655 and MG1655 Δ*mhpR* as well as BW25113 and BW25113 *mhpR::km* strains. Cells were cultivated as described above, and the total number of colony-forming units at pH 3 was counted after overnight incubation. All experiments were performed in biological replicates (*n* ≥ 6), and the error bars represent standard deviations of the mean. Significance was evaluated by performing a one-way ANOVA test followed by Bonferroni’s multiple comparisons test to compare log-transformed numbers of surviving cells (ns, not significant and ****P < 0.0001). (**D**) Schematic representation of the genomic *flhDC* locus and the distance between the *flhD* start codon and the inserted IS5 element. The presence of the IS5 sequence was determined by colony PCR and sequencing.

Nevertheless, as previously shown (12) and reconfirmed here again, we observed a strong phenotype of *E. coli* BW25113 *mhpR::km,* a knockout mutant from the Keio collection in an acid shock experiment (Fig. 2B and C). Based on this result, which somewhat contradicted all the results obtained with *E. coli* MG1655 described above, we decided to construct an MG1655 Δ*mhpR* mutant. We performed the acid shock assay (survival of cells at pH 3 for 1 h) and did not detect any significant difference between the MG1655 wild type and the Δ*mhpR* mutant (Fig. 2A and C).

To determine why an acid shock phenotype associated with the knockout of *mhpR* is exclusively detectable in *E. coli* BW25113 but not in MG1655, we searched for strain-specific differences between the two strains. It is important to note that some BW25113 mutants from the Keio collection have IS elements or point mutations in the regulatory region upstream of *flhDC*, which cause upregulation of motility genes (28). Therefore, we sequenced the corresponding region of the BW25113 *mhpR::km* mutant and found an IS5 element in the *flhDC* promoter (Fig. S2). The IS5 element was integrated 516 bp upstream of the *flhDC* start codon (Fig. 2D).

### Upregulation of motility and chemotaxis genes is not associated with MhpR

**Fig 3:**
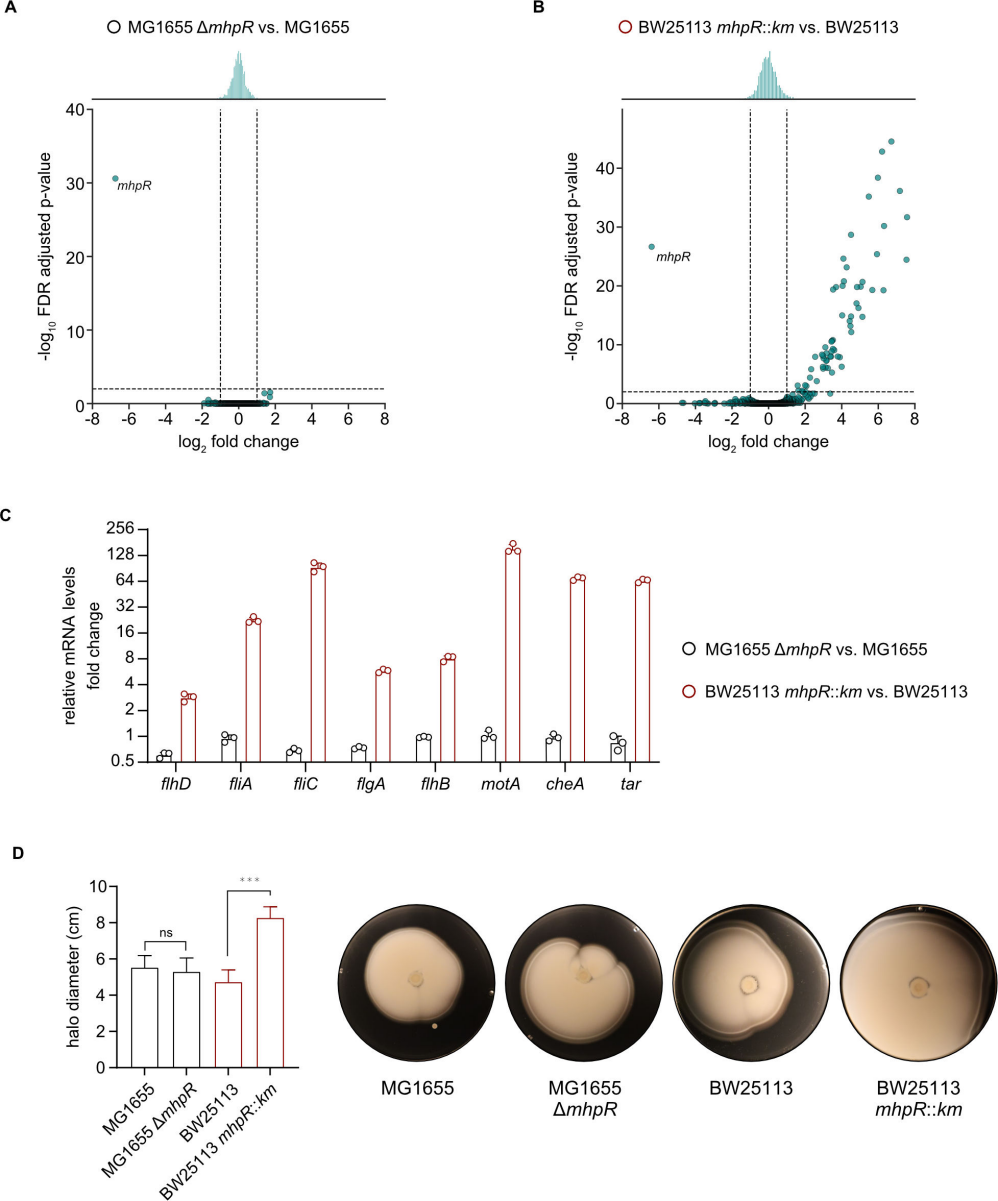
Motility and chemotaxis genes are upregulated in *E. coli* BW25113 *mhpR::km* but not in MG1655 ∆*mhpR*. (**A and B**) Volcano plots illustrating differential mRNA levels determined by RNA-Seq for MG1655 Δ*mhpR* compared to MG1655 (**A**) and BW25113 *mhpR::km* compared to BW25113 (**B**). Cells were grown to an OD_600_ of 0.5 in LB pH 7.6 before being shifted to LB pH 5.8 for 15 min and LB pH 4.4 for another 15 min. Dashed lines indicate log_2_ fold change values of +1 or −1 and *P*-adjusted values of 0.01. (**C**) Verification of a selection of differentially expressed genes in BW25113 *mhpR::km* compared to BW25113 at pH 4.4 by RT-qPCR. Cells were cultivated as described above. Fold change values were calculated relative to MG1655 or BW25113 and normalized using *secA* as a reference gene. Error bars indicate the standard deviation of independent biological replicates (*n* = 3). (**D**) Soft agar swim assay to evaluate the strains described in panels A–C. Overnight cultures normalized to an OD_600_ of 1 were spotted on LB soft agar [0.3% (wt/vol)] plates and incubated for 16 h. Halo diameters were measured, and all experiments were performed in biological replicates (*n* ≥ 4). Error bars represent standard deviations of the mean, and significance was evaluated by performing a one-way ANOVA test followed by Bonferroni’s multiple comparisons test (ns, not significant and ***P < 0.001). Representative images are shown.

We then conducted an RNA-Seq experiment to elucidate whether differentially expressed genes (DEGs) are detectable in *mhpR* mutants or whether the IS5 insertion 516 bp upstream of the *flhDC* start codon (Fig. 2D) leads exclusively to the induction of chemotaxis and motility genes. To this end, we compared the transcriptomes of *E. coli* MG1655 Δ*mhpR* and BW25113 *mhpR::km* under severe acid stress conditions (pH 4.4) with the respective parental strains. We did not detect differentially expressed genes (fold change > 2, FDR-adjusted *P*-value < 0.01) between MG1655 and MG1655 Δ*mhpR,* with the exception of the deleted *mhpR* gene (Fig. 3A).

In contrast, 62 genes were differentially expressed in the *mhpR* mutant from the Keio collection (Fig. 3B). Almost all upregulated DEGs in BW25113 *mhpR::km* were related to motility and chemotaxis (Table 1) and are part of the FlhDC regulon (13, 14). This result indicates that the IS5 insertion upstream of *flhDC* leads to the induction of motility genes. The only DEGs in BW25113 *mhpR::km* that were not related to flagellar biosynthesis and chemotaxis were *mhpA* and *mhpB* (Table 1). However, it has already been described (37) that the *mhpC* gene in strain BW25113 contains an IS30 element, which leads to a strong expression of *mhpCDEF* and probably to the deregulation of the expression of *mhpAB* in the absence of *mhpR*.

**TABLE 1:** Top 20 genes with increased mRNA levels in BW25113 *mhpR::km* compared to BW25113, sorted in descending order by RNA-Seq fold change values

Gene name	Fold change	*P*-adjust	Annotation
*motA*	190.27	2.12 × 10^−32^	Motility protein A
*tar*	186.37	3.58 × 10^−25^	Methyl-accepting chemotaxis protein
*tap*	144.98	7.40 × 10^−37^	Methyl-accepting chemotaxis protein—dipeptide-sensing
*motB*	105.21	2.90 × 10^−45^	Motility protein B
*yhjH*	79.58	6.55 × 10^−31^	Cyclic di-GMP phosphodiesterase
*fliC*	77.91	5.53 × 10^−20^	Flagellar filament structural protein
*cheA*	73.86	1.49 × 10^−43^	Chemotaxis protein
*mhpB*	62.98	4.13 × 10^−39^	3-Carboxyethylcatechol 2,3-dioxygenase
*cheW*	60.85	4.06 × 10^−26^	Chemotaxis protein
*cheB*	51.00	5.08 × 10^−20^	Protein-glutamate methylesterase/protein glutamine deamidase
*mhpA*	44.64	6.71 × 10^−36^	3-(3-hydroxyphenyl)propanoate hydroxylase
*flxA*	35.15	2.11 × 10^−21^	Qin prophage, PF14282 family protein
*tsr*	35.05	1.80 × 10^−15^	Methyl-accepting chemotaxis protein—serine-sensing
*fliS*	32.82	1.38 × 10^−20^	Flagellar biosynthesis protein
*fliT*	30.18	5.67 × 10^−17^	Flagellar biosynthesis protein
*cheY*	28.51	1.58 × 10^−20^	Chemotaxis protein
*ycgR*	28.03	9.35 × 10^−18^	Flagellar brake protein
*fliE*	22.97	7.04 × 10^−13^	Flagellar protein
*yjcZ*	22.81	1.59 × 10^−15^	Regulator of diguanylate cyclase
*fliD*	22.77	2.06 × 10^−29^	Flagellar filament capping protein

To confirm the RNA-Seq results, we tested a representative selection of DEGs (Table 1) by RT-qPCR. As expected, genes encoding flagella components (*fliA*, *fliC*, *flgA*, *flhB*, and *motA*) and chemotaxis proteins (*cheA* and *tar*) showed enriched mRNA levels in BW25113 *mhpR::km* but not in MG1655 Δ*mhpR* (Fig. 3C). In parallel, we tested the motility of the two strains on soft agar [0.3% (wt/vol)] plates. In agreement with the RNA-Seq data (Fig. 3B), BW25113 *mhpR::km* showed significantly increased spreading (*P* < 0.001) compared to MG1655 Δ*mhpR* (Fig. 3D).

### The presence or absence of an IS element upstream of *flhDC*, but not MhpR, determines survival under severe acid stress

**Fig 4:**
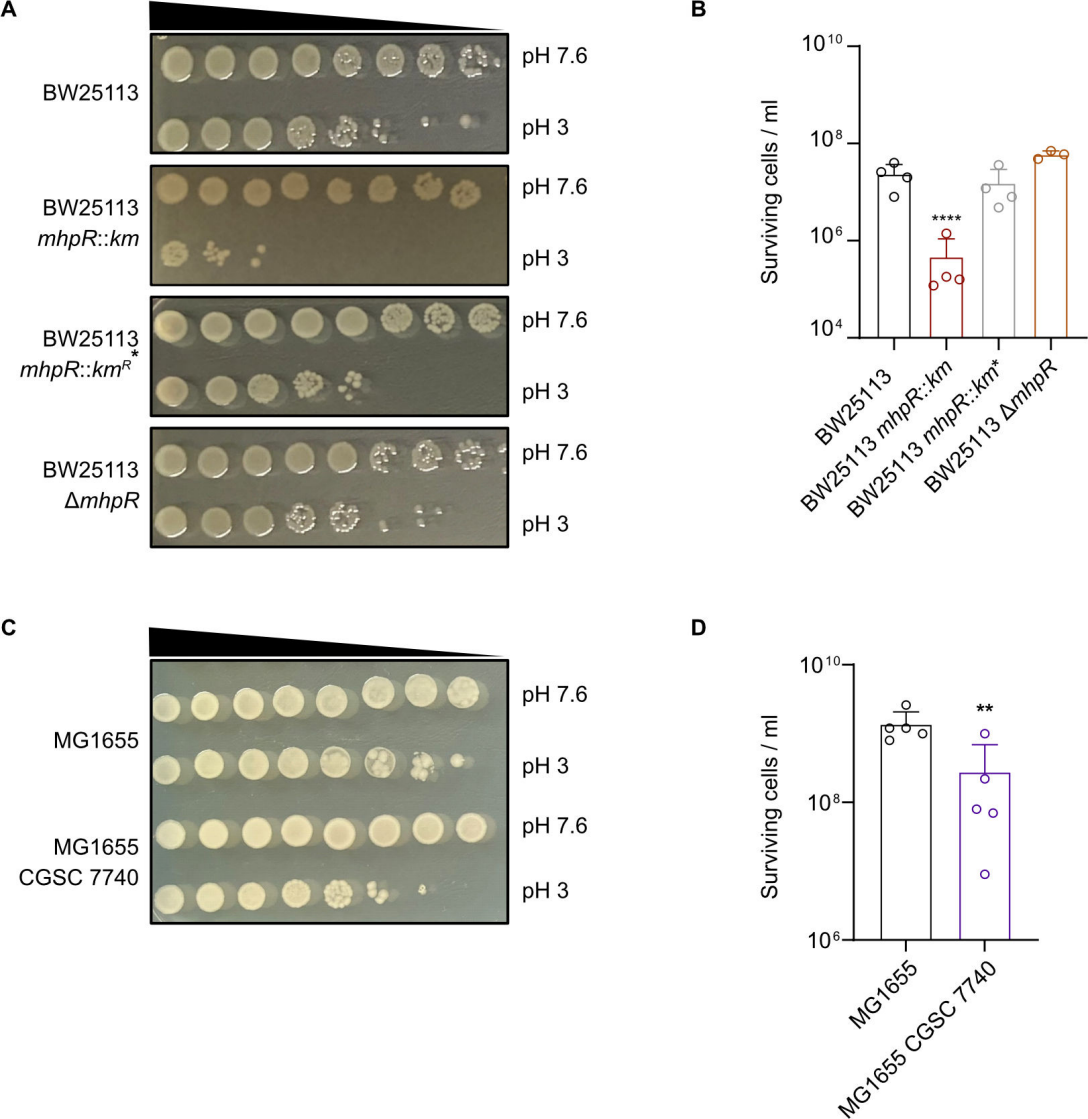
The presence or absence of insertion elements upstream of *flhDC* determines survival under severe acid stress. (**A and C**) Acid shock assays to evaluate the survival of *E. coli* BW25113, BW25113 *mhpR::km*, BW25113 mhpR::km*, BW25113 Δ*mhpR*, MG1655, and MG1655 CGSC 7740 at pH 3. Cells were grown in LB pH 7.6 to OD_600_ = 0.5 and cell numbers were adjusted to 10^9^/mL. Cultures were split and then either grown at pH 7.6 or stepwise stressed (15 min pH 5.8 and 15 min pH 4.4) before being exposed to LB pH 3 for 1 h. Cultures were serially diluted by a factor of 10 in 1× PBS and plated on LB agar plates. Images were taken after overnight incubation. (**B and D**) Quantitative assessment of acid shock survival of *E. coli* BW25113, BW25113 *mhpR::km*, BW25113 mhpR::km*, BW25113 Δ*mhpR*, MG1655, and MG1655 CGSC 7740. Cells were cultivated as described in panel A, and the total number of colony-forming units at pH 3 was counted after overnight incubation. All experiments were performed in biological replicates (*n* ≥ 3), and error bars represent standard deviations of the mean. Significance was evaluated by performing a one-way ANOVA test followed by Bonferroni’s multiple comparisons test (**B**) or an unpaired *t*-test (**D**) to compare log-transformed numbers of surviving cells (**P < 0.01 and ****P < 0.0001).

To evaluate whether the presence of the motility-activating IS5 element is indeed the cause of reduced survival under severe acid stress, we removed the IS5 element from the *flhDC* promoter of BW25113 *mhpR::km*. We found that the strain obtained from the Keio collection (29) contained a mixture of cells with and without IS5 insertion in the *flhDC* regulatory region (Fig. S3). By re-streaking this strain several times on LB agar plates and screening individual colonies, we obtained a BW25113 *mhpR* mutant without IS5 insertion in the *flhDC* promoter (Fig. S3) and designated this strain as BW25113 mhpR::km*. As expected, removal of the IS element from the *flhDC* regulatory region restored the motility of BW25113 back to wild-type levels (Fig. S4). Similar to MG1655 Δ*mhpR*, BW25113 mhpR::km*, with the restored *flhDC* regulatory region, showed no differential acid shock survival compared to the wild type (Fig. 4A and B). Furthermore, we constructed another *mhpR* knockout in BW25113 via double homologous recombination, which also contains the native *flhDC* promoter locus (BW25113 Δ*mhpR*). This strain also showed no reduced survival in the acid shock assay (Fig. 4A and B). These findings indicate that survival under acid shock is not affected by MhpR but depends directly on the presence or absence of an IS element in the intergenic region between *flhDC* and *uspC*.

Considering that the removal of motility-activating insertions improved the survival of *E. coli* under severe acid stress, we wondered whether we could provoke the reverse phenomenon by introducing an IS element into a strain with a native *flhDC* promoter. Notably, variations in terms of the presence of IS elements upstream of *flhDC* have been observed in different *E. coli* K-12 MG1655 wild-type strains (25). As shown in Fig. S2, the MG1655 wild-type strain used in our laboratory does not harbor IS elements in the *flhDC* promoter and is correspondingly less motile (Fig. 3D). In contrast, another sequenced MG1655 wild-type version (CGSC 7740) contains an IS element in the *flhDC* promoter and is highly motile (25). We thus ordered this strain, detected the presence of the IS element (Fig. S5), and confirmed that motility is indeed significantly increased (Fig. S6). As expected, acid shock survival rates of MG1655 CGSC 7740 were significantly lower (Fig. 4C and D), suggesting that the IS insertion increased motility at the cost of acid tolerance.

### Survival under severe acid stress and *flhDC* expression levels are inversely correlated

**Fig 5:**
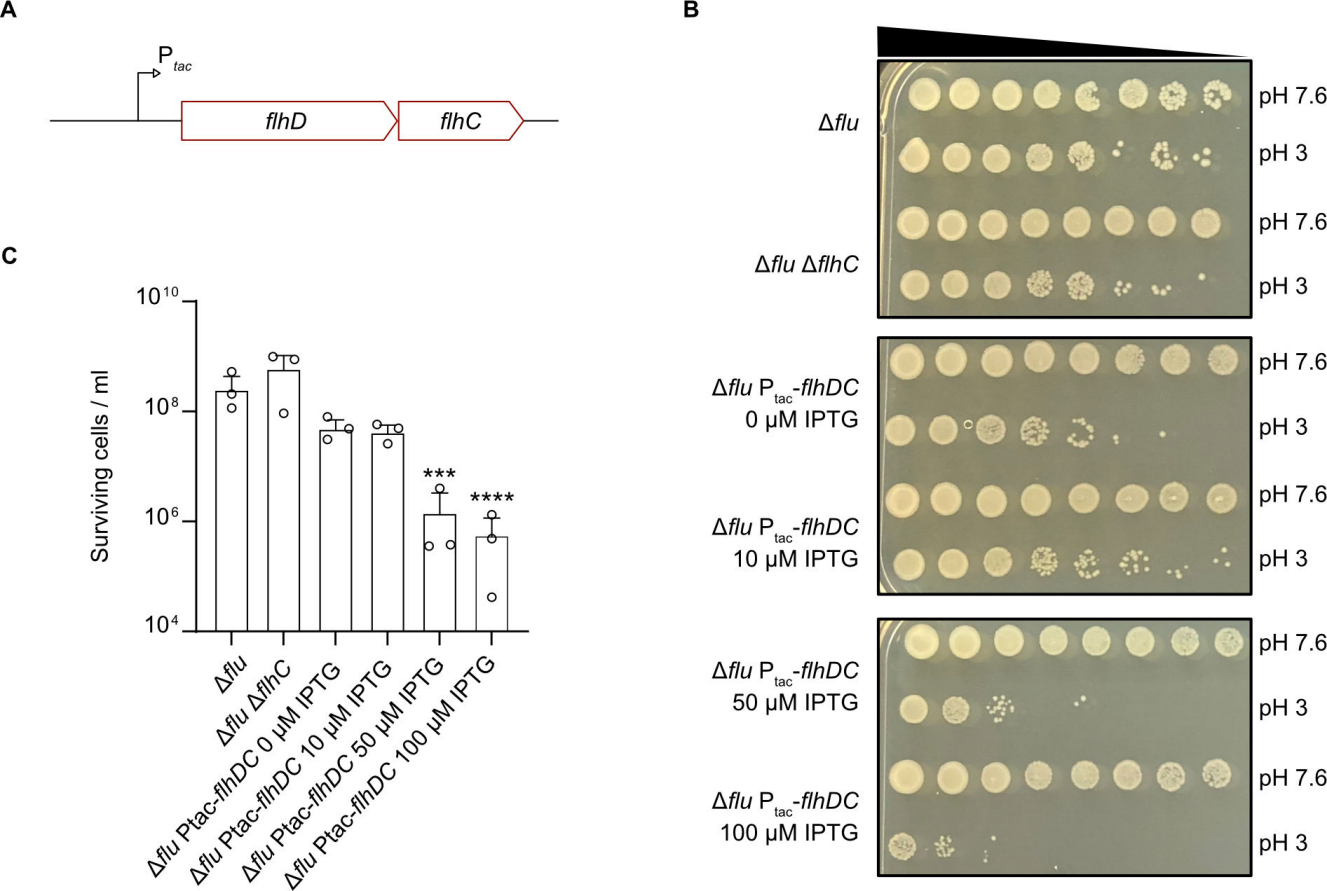
Survival under acid shock and the level of *flhDC* induction are inversely related. (**A**) Schematic representation of the *flhDC* locus with a replacement of the canonical *flhDC* promoter by an IPTG-inducible P*_tac_* promoter. (**B**) Acid shock assays to evaluate the survival of *E. coli* Δ*flu*, Δ*flu* Δ*flhC*, and Δ*flu* P_tac_-*flhDC* strains supplemented with different concentrations of IPTG. Cells were grown in LB pH 7.6 containing the indicated IPTG concentrations to OD_600_ = 0.5 and cell numbers were adjusted to 10^9^/mL. Cultures were split and then either grown at pH 7.6 or stepwise stressed (15 min pH 5.8 and 15 min pH 4.4) before being exposed to LB pH 3 for 1 h. Cultures were serially diluted by a factor of 10 in 1× PBS and plated on LB agar plates. Images were taken after overnight incubation. (**C**) Quantitative assessment of acid shock survival of *E. coli* Δ*flu*, Δ*flu* Δ*flhC*, and Δ*flu* P_tac_-*flhDC* strains supplemented with different concentrations of IPTG. Cells were cultivated as described in panel B, and the total number of colony-forming units at pH 3 was counted after overnight incubation. All experiments were performed in biological replicates (*n* = 3), and error bars represent standard deviations of the mean. Significance was evaluated by performing a one-way ANOVA test followed by Bonferroni’s multiple comparisons test to compare log-transformed numbers of surviving cells (***P < 0.001 and ****P < 0.0001).

Due to the inherent capacity of IS elements for genomic transpositions, we cannot exclude the possibility of loss or gain of IS elements upstream of *flhDC* during our experimental procedures. To circumvent the issue of IS transposition and gain the ability to fine-tune *flhDC* expression levels over a wide range, we next used an MG1655 strain in which the regulatory *flhDC* region was chromosomally replaced by a synthetic IPTG-inducible P*_tac_* promoter (Fig. 5A) (38). This strain contains a *flu* deletion to minimize cell aggregation (38). Intriguingly, we observed an inverse correlation between the supplemented IPTG concentrations, i.e., FlhDC levels, and the number of cells that survived the acid shock (Fig. 5B). The addition of 10 µM IPTG corresponds to the native *flhDC* expression level (V. Sourjik, personal communication), and no decrease in survival after acid shock was observed under this condition (Fig. 5B and C). On the other hand, overexpression of *flhDC* as a result of the addition of 50 or 100 µM IPTG led to a significant reduction in survival (Fig. 5C). Of note, neither the deletion of *flhC* nor the absence of an inducer for the P*_tac_* promoter (0 µM IPTG) had any effect on acid tolerance compared to the Δ*flu* reference strain (Fig. 5B and C).

**Fig 6:**
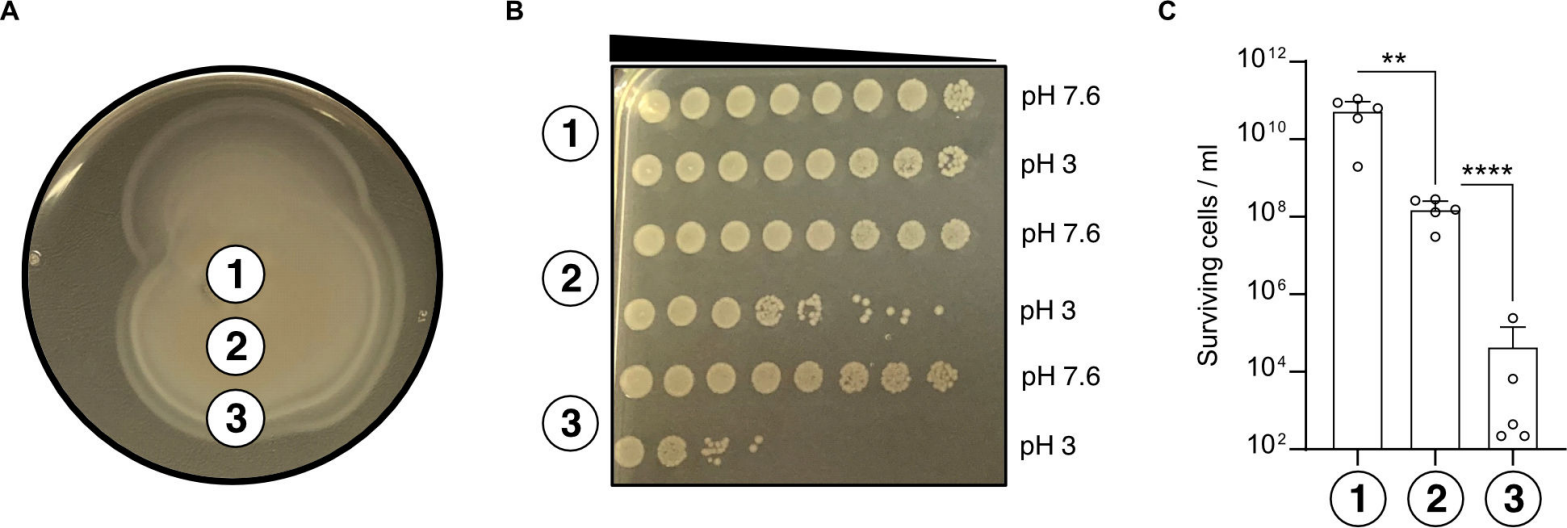
Subpopulations from swim agar plates have different abilities to survive acid shock. (**A**) Illustration of the MG1655 radial expansion on a soft agar [0.3% (wt/vol)] plate. Three microliters of exponentially grown MG1655 cultures were spotted on soft agar plates and incubated for 16 h. The labeled circles indicate the positions where the cells were collected (1, center; 2, intermediate; and 3, edge) and subsequently exposed to acid shock. (**B**) Acid shock survival assays of samples taken as described in panel A. Cells were punched out from soft agar plates using P1000 pipette tips and exposed to either LB pH 7.6 or 3 for 1 h. Cultures were serially diluted by a factor of 10 in 1× PBS and plated on LB agar plates. Images were taken after overnight incubation. (**C**) Quantitative assessment of acid shock survival of cells collected from sampling positions described in panel A. Cells were cultivated as in panels A and B, and the total number of colony-forming units at pH 3 was counted after overnight incubation. All experiments were performed in biological replicates (*n* = 5), and error bars represent standard deviations of the mean. Significance was evaluated by performing a one-way ANOVA test followed by Bonferroni’s multiple comparisons test to compare log-transformed numbers of surviving cells (**P < 0.01 and ****P < 0.0001).

To further explore the inverse correlation between acid tolerance and the degree of motility/flagellation, we isolated subpopulations from soft-agar plates and subjected them to severe acid stress. As described previously (39), we sampled MG1655 wild type grown on soft agar [0.3% (wt/vol)] from three different halo positions with increasing distance to the center (1, center; 2, intermediate; and 3, edge) (Fig. 6A). After exposure to acid shock, the cells collected from the center showed very high acid survival rates (Fig. 6B), even exceeding the values usually observed for *E. coli* MG1655 in liquid culture. Strikingly, cells from the intermediate position and especially those from the edge of the halo were characterized by a significantly lower survival rate (Fig. 6C). It should be noted that the tested subpopulations are in different growth stages and that the extreme resistance of the subpopulation from the center overlaps with the adaptation to the stationary phase (40). In conclusion, (hyper)flagellation mediates a survival deficit in very low pH habitats.

## DISCUSSION

The RNA-Seq and Ribo-Seq data from our previous study revealed that the MhpR synthesis levels increased by about one order of magnitude at pH 4.4 and that survival of an *mhpR* mutant from the Keio collection was impaired under severe acid stress (12). Here, we confirmed a higher transcript level of *mhpR* under severe acid stress but found no effects of the transcription factor itself, the regulated 3HPP/PP-dependent catabolic operon, or the cinnamic acid derivatives on acid tolerance.

Thus, despite the upregulation of MhpR at pH 4.4, MhpR does not confer a survival benefit for *E. coli* when challenged with acid at pH 3, at least not in our experimental setup. It is important to note that we could not detect MhpR-inducers such as 3HPP or PP in pH-neutral or acidified LB media via LC-MS. Therefore, we cannot neglect that MhpR and the degradation of cinnamic acid derivatives are useful in nature. PP and 3HPP are present in the human gastrointestinal tract due to the metabolism of aromatic amino acids and plant-derived flavonoids (35), so *E. coli* is exposed to these molecules under natural conditions. Indeed, the expression of *mhp* catabolic genes in enterohemorrhagic *E. coli* (EHEC) increases during growth in the cecal contents of human gut microbiota-associated rats (41). Under these conditions, the cinnamic acid derivatives 3HPP and PP are utilized as carbon sources (30). *mhpR* expression was also significantly increased in EHEC upon infection of mice, and an *mhpR* mutant was outcompeted by a wild type during colonization of the mouse intestine (42). However, our RNA-Seq analysis of *E. coli* K12 provided no evidence that MhpR regulates a gene important for acid tolerance.

BW25113 mutants harboring both, an IS5 insertion in the *flhDC* promoter and an *mhpR* deletion, showed an acid shock phenotype, whereas the BW25113 Δ*mhpR* and mhpR::km* strains with the native *flhDC* upstream region did not (Fig. 4). This indicated that the motility-inducing IS5 insertion is the cause of the observed phenotype in the BW25113 *mhpR::km* strain (Fig. 2). Similar IS insertions and point mutations were described in other mutants of the Keio collection. In fact, 49 of 71 tested strains had mutations in the upstream region of *flhDC,* and as a result, showed increased expression of genes involved in flagellar biosynthesis (28). It is known that the integration of IS elements in the *flhDC* promoter is favored under resting (nonshaking) conditions (28), and mutants from the Keio collection were constructed by culturing bacteria overnight without shaking (29). Moreover, it is hypothesized that IS insertion in this genomic locus is triggered by the cellular environment and depends on whether the encountered conditions permit motility (26).

Our results clearly indicate that IS5 integration upstream of *flhDC* reduces population survival under acid shock in a manner that is inversely correlated with *flhDC* expression levels (Fig. 5). Likewise, acid shock tolerance was dependent on the location of swimming cells on the soft-agar surface, and cells at the edge of the halo could barely survive at pH 3 (Fig. 6). As indicated by RNA-Seq and RT-qPCR, the reason for the reduced acid shock survival was increased flagellar and motility gene expression (Fig. 3). However, it remains to be clarified why increased flagellation and/or motility reduces survival under severe acid stress. It appears plausible that hypermotile cells have increased energy demand resulting from flagellar synthesis, including the motor and filaments (43); the cost of synthesizing and operating the flagella accounts for up to 3.5% of the total cell energy (44–46). Therefore, cells with IS insertions upstream of *flhDC* are characterized by a reduced growth rate (26). Furthermore, a high number of rotating flagella affects not only the integrity of the membrane but also causes a high flux of protons back into the cytoplasm, which might be disadvantageous at low pH.

Under severe acid stress (pH 4.0–4.5), flagella are rapidly shed (47, 48), chemotaxis to attractants ceases, and the bacteria are no longer motile (49). On the other hand, several studies reported an increased expression of motility genes under mild acid stress (10–12). We propose that under mildly acidic conditions, *E. coli* uses an escape strategy to migrate to environments with optimal pH. Under severe acidic conditions, *E. coli* abandons the escape strategy, switches to an energy-saving mode, and also prevents the flux of protons into the cytoplasm. Considering that motility genes are still induced at pH 5.0 (10), we hypothesize that the inversion point at which motility stops to be beneficial is between pH 5.0 and 4.4.

Motility-activating mutations in the regulatory region of *flhDC* have already been shown to represent a trade-off between growth and biofilm formation (26). Strains containing IS5 upstream of *flhDC* produced more biofilms at the expense of overall growth (26). In this study, we present another trade-off associated with the presence of IS elements, namely the relationship between acid tolerance and the expression of motility genes. It is important to note that the transposition of IS elements can lead to phenotypic heterogeneity within bacterial populations. For example, the phenotypic variability of cells in a biofilm increased as a function of the frequency of IS5 insertions upstream of *flhDC* (26). Moreover, IS-mediated motility heterogeneity within a biofilm was beneficial for bacteria to increase biofilm mass (50). Also, in this study, we found a heterogeneous population of strain CGSC 7740 MG1655 with respect to cells carrying an IS element upstream of *flhDC* (Fig. S5). We have already shown the advantage of phenotypic heterogeneity under acid stress for the three major AR systems using a triple fluorescent reporter strain that enables bet-hedging and division of labor in *E. coli* (51). It is possible that the cytoplasmic membrane protein HdeD represents a link between the acid resistance Gad system and flagellar synthesis in *E. coli*. The expression of *hdeD* is controlled by GadE and HdeD represses the flagella biosynthesis via LrhA (52). Heterogeneous distribution of HdeD in the *E. coli* population (53) and heterogeneous expression of flagella in *Salmonella* are known (54, 55). Using single-cell RNA-Seq (M3-Seq), an acid-resistant subpopulation was found in stationary phase *E. coli* cells (56). According to the results of this study, the transposition of IS elements could be another important factor leading to heterogeneity under acid stress, and it is tempting to speculate that there might also be a motile subpopulation in stationary phase *E. coli* cells (56). This assumption is supported by the data collected here, which suggest an anti-correlation between motility and acid tolerance mediated by IS integrations upstream of *flhDC*. Of note, motility was also found to be correlated with oxygen availability (57). Given the different levels of acidity in the gastrointestinal tract, with a pH of <2 in the stomach (5, 58) and ~pH 6 in the duodenum (59, 60), it would certainly be advantageous for an *E. coli* population to diversify into a motile and an acid-tolerant subpopulation. Indeed, colonization of the mouse intestine was affected by the presence of IS upstream of *flhDC* in *E. coli* MG1655, indicating niches where motility is advantageous (61). In light of the presence of different intestinal niches where either acid tolerance or motility is beneficial, IS transposition upstream of *flhDC* could be crucial to ensure that *E. coli* is able to colonize both niches by dividing into a motile and an acid-tolerant subpopulation.

Taken together, this study demonstrates that the presence or absence of motility-activating mutations upstream of the master regulatory genes *flhDC* is important for *E. coli* to survive severe acid stress. The FlhDC levels are found to be anticorrelated with survival at pH 3, and motile subpopulations exhibit extremely low acid tolerance. These findings highlight a fitness trade-off between acid tolerance and motility and suggest an IS-mediated differentiation of *E. coli* into motile and acid-tolerant subpopulations.

## MATERIALS AND METHODS

### Bacterial strains and growth conditions

*E. coli* MG1655 (62) and BW25113 strains (63) and plasmids used in this study are listed in Tables S2 and S3. Cells were grown in LB medium (10 g/L tryptone, 5 g/L yeast extract, and 10 g/L NaCl) and incubated aerobically in a rotary shaker at 37°C. When appropriate, media were supplemented with 15 µg/mL gentamicin or 50 µg/mL kanamycin. For RNA-Seq experiments, the pH of the medium was adjusted by the direct addition of 5 M HCl to growing cultures, as described in Schumacher et al. (12).

### Plasmid construction

Molecular methods were performed according to standard protocols or according to the manufacturer’s instructions. Kits for the isolation of plasmids and the purification of PCR products were purchased from Süd-Laborbedarf. Enzymes and HiFi DNA Assembly Master Mix were purchased from New England Biolabs. To construct the reporter plasmids pBBR1-MCS5-P*_mhpR_*_:*lux*_ and P*_mhpABCDFE_*_:*lux*_, 200 nt of the upstream regions of the respective genes were amplified by PCR using primers KSO-0169–KSO-0172 and MG1655 genomic DNA as a template. For the construction of the pNPTS-R6KT-Δ*mhpR* plasmid, 1,000 nt upstream and downstream of the *mhpR* coding region were amplified. After purification, fragments were assembled into PCR-linearized pBBR1-MCS5 or pNPTS-R6KT vectors via Gibson assembly (64). Correct insertions were verified by colony PCR and sequencing.

### Construction of chromosomal *mhpR* deletions

Construction of the marker-less in-frame deletion strains of *mhpR* in *E. coli* MG1655 and BW25113 was achieved using the suicide plasmid pNPTS138-R6KT Δ*mhpR*. The plasmid pNPTS138-R6KT Δ*mhpR* was introduced into *E. coli* MG1655 and BW25113 by conjugative mating using *E. coli* ST18 (65) as a donor in LB medium containing 50 µg/mL 5-aminolevulinic acid (Ala). Single-crossover integration mutants were selected on LB plates containing kanamycin but lacking Ala. Single colonies were grown over a day without antibiotics and plated onto LB plates containing 10% (wt/vol) sucrose and lacking NaCl to select for plasmid excision. Kanamycin-sensitive colonies were investigated in terms of *mhpR* deletion by colony PCR using primers up- and downstream of the site of the insertion. Deletion of *mhpR* was verified by sequencing.

### Acid shock assay

Acid shock assays were conducted as described (12). Briefly, cells were incubated at 37°C in LB medium (pH 7.6) until an OD_600_ of 0.5 was reached. Upon adjustment to an OD_600_ of 1, cells were either grown at pH 7.6 throughout the experiment or stepwise pH adjusted (15 min pH 5.8 and 15 min pH 4.4) before being shifted to pH 3 for 1 h. Next, samples were serially diluted by a factor of 10 in 1× PBS and plated on LB agar plates. Colony-forming units were counted the next day, and significance was evaluated by performing a one-way ANOVA test followed by Bonferroni’s multiple comparisons test.

To evaluate acid shock survival of MG1655 Δ*flu* P*_tac_-flhDC*, cells were grown as described above. All media were supplemented with either 0, 10, 50, or 100 µM IPTG, throughout the experiment.

Acid shock survival of MG1655 subpopulations obtained from different sampling areas of soft agar plates was evaluated using 3 µL of cells grown to OD_600_ of 0.4. Cells were spotted on soft agar plates [0.3% (wt/vol)] and incubated for 16 h at 37°C as described by Bubendorfer and colleagues (39). Subsequently, cells from three different sampling areas (1, center; 2, intermediate; and 3, edge) (Fig. 6A) were punched out using a P1000 pipette tip and immediately resuspended in either 1 mL LB pH 7.6 or pH 3. Serial dilution and data analysis were conducted as described above.

### RNA-Seq analysis

Biological triplicates of *E. coli* MG1655 wild type or Δ*mhpR*, as well as BW25113 wild-type or *mhpR::km* cells, were inoculated to a starting OD_600_ of 0.05 from overnight cultures and grown in 200 mL of unbuffered LB medium (pH 7.6) until an OD_600_ of 0.5 was reached. Cultures were shifted first for 15 min to LB pH 5.8 and subsequently for 15 min to LB pH 4.4. pH shifts were achieved by adding 5 M HCl directly to growing cultures. pH values before and after pH shifts, as well as final optical densities, were monitored (Table S1). Upon exposure to pH 4.4, 1.6 mL of stop mix [95% (vol/vol) ethanol and 5% (vol/vol) phenol] was added to 8 mL aliquots of the respective cultures to terminate ongoing transcription and translation. Samples were flash frozen in liquid nitrogen and stored at −80°C until RNA isolation. Cells were pelleted (3,000 × *g*, 15 min, 4°C), and total RNA was isolated using the miRNeasy Mini Kit (Qiagen) in combination with the RNase-Free DNase Set (Qiagen). RNA samples were evaluated in terms of integrity using an RNA 6000 Nano Kit (Agilent) and quantified using a Qubit RNA HS Assay Kit (Invitrogen). Ribosomal RNA depletion was performed using the NEBNext rRNA Depletion Kit for bacteria (NEB), and directional cDNA libraries were prepared using the NEBNext Ultra II Directional RNA Library Prep Kit for Illumina (NEB). cDNA library quality was evaluated using a High Sensitivity DNA Kit (Agilent). Finally, cDNA libraries were sequenced using a NextSeq 1000 machine (Illumina) in single-read mode with a 60 bp read length.

The demultiplexed read files in fastq format were imported into the CLC Genomics Workbench v20.0.4 (Qiagen) and trimmed for quality and adaptors. Reads were mapped to the *E. coli* MG1655 and BW25113 reference genomes (NCBI accession numbers: NC_000913.3 and CP009273.1) using the “RNA-Seq Analysis” tool with default parameters. Reads that mapped to annotated genes were normalized (reads per kilobase per million reads mapped rpkm) and transformed (log_2_). Low-expression transcripts were filtered out, and we focused our analysis on genes with rpkm values ≥ 5 in at least one replicate. Differential expression was evaluated using the “Empirical Analysis of DGE” tool. Genes with a fold change ≥ 2 and an FDR-adjusted *P*-value ≤ 0.01 were considered as differentially expressed. Volcano plots were created using the seaborn.jointplot function in Python 3.8.8. A comprehensive overview of all expression values is available in Tables S5 and S6.

### RNA isolation and RT-qPCR analysis

RNA isolation and RT-qPCR analysis were conducted as described (12). In brief, RNA was isolated using the miRNeasy Mini Kit (Qiagen) in combination with the RNase-Free DNase Set (Qiagen) according to the manufacturer’s instructions. A 500 ng aliquot of isolated RNA was converted to cDNA with the iScript Advanced Kit (Bio-Rad) according to the manufacturer’s instructions. Next, 1 µL of a 1:10 dilution of the cDNA samples in nuclease-free water was mixed with 5 µL of SsoAdvanced Univ SYBR Green Supermix (Bio-Rad) and 0.8 µL of 5 µM forward and reverse primers (Table S4), and the total reaction volume was adjusted to 10 µL with nuclease-free water. The mixture was dispensed in triplicates in a 96-well PCR plate (Bio-Rad) and subjected to qPCR in a Bio-Rad CFX real-time cycler. Data were analyzed according to the ΔΔCt method (66), using the *secA* gene as a reference.

### Promoter activity assay

Promoter activities of *mhpR* and *mhpABCDFE* were determined using luminescence-based reporter plasmids harboring fusions of the respective promoter regions to the *luxCDABE* genes from *Photorhabdus luminescens* encoded on a pBBR-MCS5 vector. MG1655 cells were transformed with plasmids pBBR1-P*_mhpR_*_:*lux*_ or pBBR1-P*_mhpABCDFE_*_:*lux*_. All strains were cultivated in LB medium supplemented with gentamicin overnight, and day cultures were inoculated to an OD_600_ of 0.05 in fresh LB medium (pH 7.6) and aerobically cultivated until the exponential phase (OD_600_ = 0.5). Cultures were then either shifted to LB pH 5.8 for 15 min and LB pH 4.4 for 15 min or further cultivated at pH 7.6 and supplemented with 1 mM PP (Sigma-Aldrich), 1 mM 3HPP (Fisher Scientific), or DMSO. PP and 3HPP were solved in DMSO. In the next step, cells were transferred to a 96-well plate and cultivated at 37°C in the above-mentioned media supplemented with gentamicin. Growth and bioluminescence were measured every 10 min in microtiter plates using a CLARIOstar Plus plate reader (BMG Labtech). Data are reported as relative light units in counts per second of OD_600_.

### Swim agar assay

To determine colony expansion, overnight cultures grown in LB medium were diluted in fresh LB medium and normalized to an OD_600_ of 1 before being dropped in the center of an LB soft agar plate [0.3% (wt/vol)]. After incubation at 37°C for 16 h, the diameter of the halo was measured. Significance was evaluated by performing a one-way ANOVA test followed by Bonferroni’s multiple comparisons test.

### Analysis of cinnamic acid derivatives via LC-MS

For sample preparation, 100 µL culture media was diluted with 100 µL of acetonitrile. After vigorously shaking the samples for 1 min, the samples were centrifuged at 10,000 rpm for 10 min at 10°C. The supernatants were analyzed by means of LC-MS/MS with a 5500 QTrap (AB Sciex) coupled to an ExionLC AD UPLC (AB Sciex). For chromatographic separation, a Kinetex 1.7 µm C18 100 × 2.1 mm (Phenomenex) was used with 0.1% formic acid as solvent A and acetonitrile with 0.1% formic acid as solvent B. First, 5% B was held for 0.5 min and then a linear gradient was used from 5% B to 100% B in 5 min. Afterward, the column was flushed and equilibrated to starting conditions. The separation was performed using a 400 µL/min flow rate at 40°C column oven temperature. Ions were analyzed by MS in the negative ionization mode. The spray voltage was set to −4,500 V at a source temperature of 400°C using nitrogen as collision gas. The parameters for the collision-activated dissociation were medium, curtain gas: 35 psi, ion source gas 1: 55 psi, ion source gas 2: 65 psi, entrance potential: −10 V, and the dwell time: 100 ms. The MRM (multiple reaction monitoring) transition for each compound was optimized by direct infusion of the reference standards. The MRM settings were as follows: 3-(4-hydroxyphenyl)propionic acid: 162.931 ≥ 90.9 (quantifier), declustering potential (DP) −35 V, collision energy (CE) −36 V, cell exit potential (CXP) −1 V; 162.931 ≥ 64.8 (qualifier), DP −35 V, CE −44 V, and CXP −9 V; trans-3-hydroxycinnamic acid: 164.917 ≥ 120.8 (quantifier), DP −40 V, CE −16 V, CXP −19 V; 164.917 ≥ 80.0 (qualifier), DP −40 V, CE −40 V, CXP −3 V; and hydrocinnamic acid: 148.949 ≥ 149.0 (quantifier), DP −65 V, CE −10 V, CXP −7 V; 148.949 ≥ 105.0 (qualifier), DP −65 V, CE −14 V, and CXP −5 V. Analyst 1.7. was used to acquire the data, and Multiquant 3.0.3 was used to analyze the data (both AB Sciex).

## Data Availability

The RNA-Seq raw data were deposited at Gene Expression Omnibus (GEO) under the accession number GSE260455. Additional information required to reanalyze the data reported in this paper is available from the lead contact upon request.

## SUPPLEMENTAL MATERIAL

Supplemental material spectrum.00544-24-s0001.pdf Fig. S1 to S6; Table S1 to S4.

Table S5 spectrum.00544-24-s0002.xlsx RNA-Seq results MG1655 dmhpR vs MG1655 - pH 4.4.

Table S6 spectrum.00544-24-s0003.xlsx RNA-Seq results BW25113 mhpR-km vs BW25113 - pH 4.4.

ASM does not own the copyrights to Supplemental Material that may be linked to, or accessed through, an article. The authors have granted ASM a non-exclusive, world-wide license to publish the Supplemental Material files. Please contact the corresponding author directly for reuse.
