# Supplementary material for: Motility-activating mutations upstream of flhDC reduce acid shock survival of Escherichia coli
Source: Microbiol Spectr. 2024 Apr 23;12(6):e00544-24. doi: 10.1128/spectrum.00544-24 (PMC11237407; doi:10.1128/spectrum.00544-24)
Supplement: Supplemental material — Fig. S1 to S6; Table S1 to S4. [file spectrum.00544-24-s0001.pdf]

Supplementary information

Motility-activating mutations upstream of *flhDC* reduce acid shock survival of *Escherichia coli*

Kilian Schumacher, Djanna Braun, Karin Kleigrewe, and Kirsten Jung

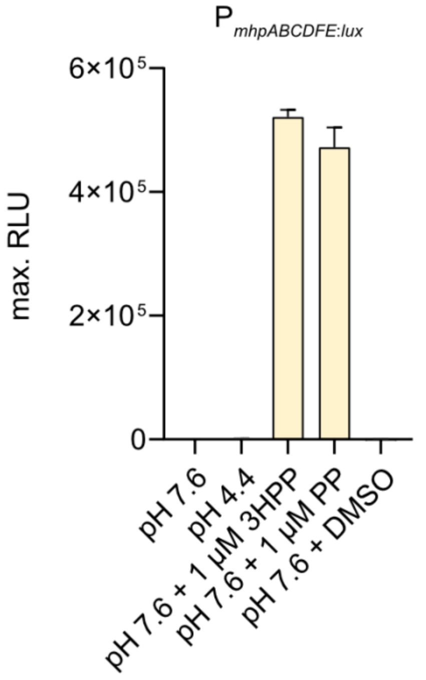

Figure S1: Promoter activity of the *mhpABCDFE* operon. *E. coli* MG1655 wild type cells were transformed with plasmid pBBR1-MCS5-$P_{mhpABCDFE:lux}$ and grown in LB medium (pH 7.6) until OD_{600} = 0.5. The medium pH was then either stepwise adjusted to 5.8 and pH 4.4, or 1 mM 3HPP, 1 mM PP, or DMSO were added. Luminescence and growth were determined every 10 min in microtiter plates using a CLARIOstar plus plate reader (BMG Labtech). Data are reported as relative light units (RLUs) in counts per second per OD_{600}, with maximal RLU shown. All experiments were performed in biological replicates (n = 3), and error bars represent standard deviations of the mean.

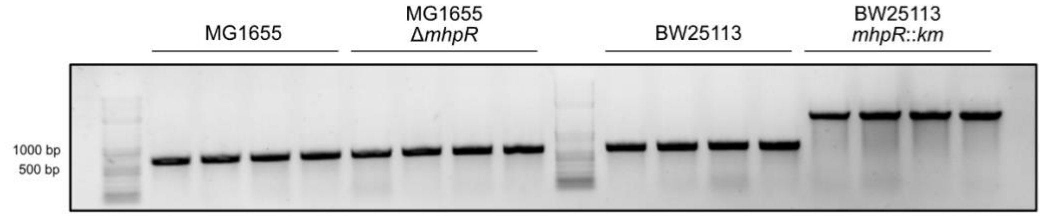

Figure S2: Transposon insertions in the promoter region of *flhDC*. The presence of IS elements in the intergenic region between *flhD* and *uspC* was verified by colony PCR using primers KSO-244 and KSO-245. The expected PCR product sizes were 767 bp in the absence of an IS element and ~2000 bp in the presence.

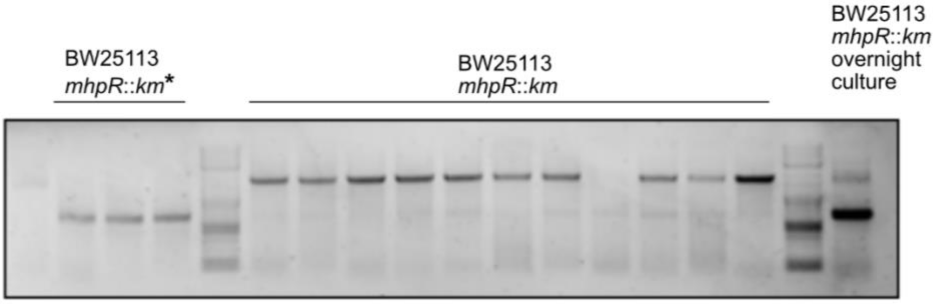

Figure S3: Verification of a BW25113 *mhpR*::*km strain without IS insertion in the promoter region of *flhDC***. BW25113 *mhpR*::*km* was streaked on LB agar plates, and single colonies were screened by colony PCR using primers KSO-244 and KSO-245 for an intact *flhDC* promoter. The expected PCR product sizes were 767 bp in the absence of an IS element and ~2000 bp in the presence.

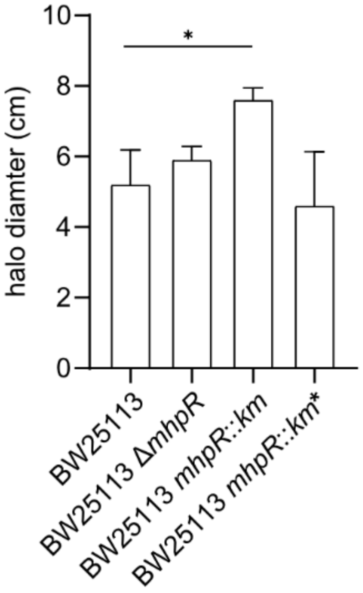

Figure S4: IS element dependent motility of BW25113. Soft agar assay evaluating the motility of BW25113 wild type, Δ*mhpR*, *mhpR*::*km*, and, *mhpR*::*km** strains. Overnight cultures normalized to an OD_{600} of 1 were spotted on LB soft agar [0.3 % (w/v)] and incubated for 16 h. Halo diameters were measured and all experiments were performed in biological replicates ($n \geq 4$). Error bars represent standard deviations of the mean and significance was evaluated by performing a one-way ANOVA test followed by Bonferroni's multiple comparisons test (* $p < 0.05$).

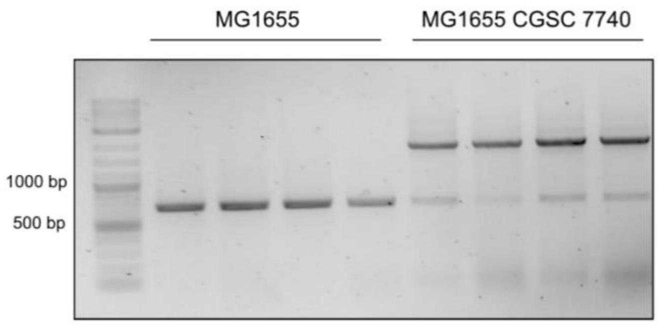

Figure S5: Alterations in the *flhDC* promoter region of two different MG1655 strains. The presence of IS elements in the intergenic region between *flhD* and *uspC* was verified by colony PCR using primers KSO-244 and KSO-245. The expected PCR product sizes were 767 bp in the absence of an IS element and ~2000 bp in the presence.

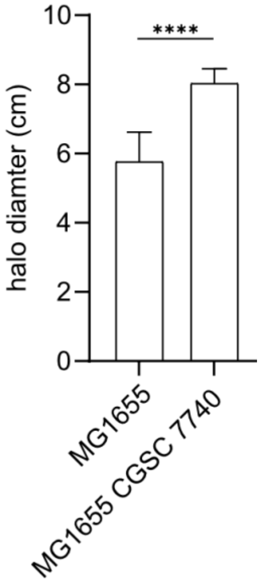

Figure S6: IS element dependent motility of MG1655. Soft agar assay evaluating the motility of MG1655 and MG1655 CGSC7740. Overnight cultures normalized to an OD_{600} of 1 were spotted on LB soft agar [0.3% (w/v)] and incubated for 16 h. Halo diameters were measured and all experiments were performed in biological replicates ($n \geq 4$). Error bars represent standard deviations of the mean and significance was evaluated by performing an unpaired t-test (**** $p < 0.0001$).

Table S1: OD_{600} and pH values determined throughout the experiment conducted for RNA-Seq. pH-shifts were initiated by direct addition of 5 M HCl to growing cultures.

	**Replicate I**				**Replicate II**				**Replicate III**			
Strain	MG1655	MG1655 Δ*mhpR*	BW25113	BW25113 *mhpR*::*km*	MG1655	MG1655 Δ*mhpR*	BW25113	BW25113 *mhpR*::*km*	MG1655	MG1655 Δ*mhpR*	BW25113	BW25113 *mhpR*::*km*
OD600 (0.5)	0.50	0.46	0.47	0.48	0.50	0.50	0.42	0.44	0.45	0.41	0.46	0.49
OD600 (end)	0.88	0.74	0.74	0.91	0.76	0.64	0.62	0.61	0.65	0.78	0.71	0.87
pH (0 min)	7.24	7.24	7.23	7.21	7.26	7.27	7.27	7.27	7.28	7.25	7.23	7.20
pH (15 min)	5.72	5.75	5.71	5.65	5.78	5.78	5.81	5.75	5.83	5.82	5.77	5.79
pH (30 min)	4.38	4.33	4.33	4.27	4.34	4.34	4.32	4.32	4.32	4.33	4.47	4.46

Table S2: *Escherichia coli* strains used in this study.

Strain	Relevant genotype or description	Reference
MG1655	K-12, F-, λ- *ilvG*-, *rfb*-50, *rph*-1	(62)
BW25113	F-, Δ(*araD*-*araB*)567, Δ*lacZ*4787(::*rrnB*-3), λ-, *rph*-1, Δ(*rhaD*-*rhaB*)568, *hsdR*514	(63)
ST18	S17 λ*pir*Δ*hemA*	(65)
MG1655 Δ*mhpR*	K-12, F-, λ- *ilvG*-, *rfb*-50, *rph*-1, Δ*mhpR*	This study
BW25113 *mhpR*::*km*	F-, Δ(*araD*-*araB*)567, Δ*lacZ*4787(::*rrnB*-3), λ-, *rph*-1 , Δ*mhpR*786::*kan*, Δ(*rhaD*-*rhaB*)568, *hsdR*514, IS5 insertion 516 bp upstream of *flhDC*	(29)
BW25113 Δ*mhpR*	F-, Δ(*araD*-*araB*)567, Δ*lacZ*4787(::*rrnB*-3), λ-, *rph*-1, Δ(*rhaD*-*rhaB*)568, *hsdR*514 Δ*mhpR*	This study
BW25113 *mhpR*::*km**	F-, Δ(*araD*-*araB*)567, Δ*lacZ*4787(::*rrnB*-3), λ-, *rph*-1, Δ*mhpR*786::*kan*, Δ(*rhaD*-*rhaB*)568, *hsdR*514	This study
MG1655 CGSC 7740	K-12, F-, λ- *ilvG*-, *rfb*-50, *rph*-1, IS insertion upstream of *flhDC*	(62)
MG1655 Δ*flu*	K-12, F-, λ- *ilvG*-, *rfb*-50, *rph*-1, Δ*flu*:.FLP	(37)
MG1655 Δ*flu* Δ*flhC*	K-12, F-, λ- *ilvG*-, *rfb*-50, *rph*-1, Δ*flu*:.FLP, Δ*flhC*:.FLP	(37)
MG1655 Δ*flu* P_{tac}-*flhDC*	K-12, F-, λ- *ilvG*-, *rfb*-50, *rph*-1, Δ*flu*:.FLP-*lacI*-P_{tac}-*flhDC*	(37)

Table S3: Plasmids used in this study.

Name	Reference
pBBR1-MCS5-P*mhpR*:*lux*	This study
pBBR1-MCS5-P*mhpABCDFE*:*lux*	This study
pNPTS-R6KT-Δ*mhpR*	This study

Table S4: Oligonucleotides used in this study.

Name	Sequence
KSO-0025	CCCGGGCTGCAGGAATTC
KSO-0026	TCTAGAGAATAGGAACTTCGGAAT
KSO-0033	ATCTGGATCCACGAATTCGC
KSO-0034	ATCCTGCAGAGAAGCTTGG
KSO-0041	CAGGAAACAGCTATGACC
KSO-0042	TGTAAAACGACGGCCAGT
KSO-0149	AGCCGCTTATCCTTTCACC
KSO-0150	TTCCTTGCGGTCTTGTTCC
KSO-0151	CAGATATTGTGCTCGGTGGTAG
KSO-0152	GACAGGTAGAAACGGGAAGAAC
KSO-0169	CGAAGTTCCTATTCTCTAGATTCAGTACCTCACGACTCGG
KSO-0170	TCGAATTCCTGCAGCCCGGGATTAATTGACATTTCTATAGTTAAAACAAC
KSO-0171	CGAAGTTCCTATTCTCTAGAATTAATTGACATTTCTATAGTTAAAACAAC
KSO-0172	TCGAATTCCTGCAGCCCGGGTTCAGTACCTCACGACTCGG
KSO-0190	GCCAAGCTTCTCTGCAGGATCCAGCAGGCGAAAATCCTG
KSO-0191	CACCATCGAATGGCGCAAAAC
KSO-0192	TTTTGCGCCATTCGATGGTGATTAATTGACATTTCTATAGTTAAAACAAC
KSO-0193	GCGAATTCGTGGATCCAGATGGCAGCACTTTGCTTAACAG
KSO-0194	CCCGCGTTTTCGCAGAAAC
KSO-0195	TTCAGTACCTCACGACTCGG
KSO-0214	GCCAAGCTTCTCTGCAGGATGAGTTGCAGCAAGCGGTC
KSO-0224	AGAGATTGAGACGCACGAAAG
KSO-0225	CCATTACAGCCGCAACAATAC
KSO-0226	TATTTGCACCTGGCTTCTCC
KSO-0227	CCGCCCATCTTCTGGATATTAC
KSO-0228	TCGCCGTATGTTGCTTACTC
KSO-0229	CCGTCAATGGTCAGGGTATTC
KSO-0230	TGAAGTGGCACAGGCAATAG
KSO-0231	TCGGCAATATCGATCCCTAAAC
KSO-0232	GGAAGCTGGCAATGTCAAAC
KSO-0233	ATAGATCGCGCAGGCTAATG
KSO-0234	GTCATTCCAATGGTGGGATTTG
KSO-0235	GGTCACCTTCGCTTTGTTTG
KSO-0236	ACTGGTTTATGACCTGGGAAC
KSO-0237	CATCGACGCCATTACACAAAC
KSO-0238	GTTAAGCTGGCAGAAACCAATC
KSO-0239	ATCGTCAACGCGGGAATC
KSO-0240	ACCTGAACAACACCACTACC
KSO-0241	GATCTGCGCTTTCGACATATTG
KSO-0242	CGGTACGTCGCTTTGAATTTATG
KSO-0243	GATTAGGCAGCACTTTGCTTAAC
KSO-0244	GTTTCACCGCACCCCGTG
KSO-0245	GGAGAAACGACGCAATCCCAAC
